# 620. Identifying the Role for a Pharmacist on an Outpatient Parenteral Antimicrobial Therapy (OPAT) Team in an Academic Teaching Hospital

**DOI:** 10.1093/ofid/ofab466.818

**Published:** 2021-12-04

**Authors:** Garret H Hino, Jacinda Abdul-Mutakabbir, Norman Hamada, Anna Zhou, Karen K Tan

**Affiliations:** 1 Loma Linda University, Honolulu, Hawaii; 2 Loma Linda University School of Pharmacy, Redlands, California; 3 Loma Linda University Medical Center, Los Angeles, California

## Abstract

**Background:**

Outpatient parenteral antimicrobial therapy (OPAT) is currently an emerging practice to continue effective treatment after hospital discharge for patients requiring parenteral (IV) treatment. Pharmacists can collaborate with outpatient services like home infusion services to allow for safe administration and monitoring of IV antibiotics. The role of pharmacists in an OPAT team has been shown to improve patient outcomes such as optimizing antimicrobial therapy and reducing hospital length of stay and readmissions. We sought to define the utility of an OPAT pharmacist at an academic teaching hospital that currently does not have an OPAT service.

**Methods:**

Patients receiving IV therapy via home infusion from 1/4/21 to 3/4/21 were screened for inclusion and excluded if antimicrobials were not prescribed. Infection characteristics and antimicrobial therapy were recorded. Interventions on day of and after discharge were noted. Duration of therapy (DOT) was calculated by the difference between start and stop dates of appropriate antibiotics. Discharge delays due to OPAT-related reasons were recorded. Continuous data are expressed as median (IQR). Categorical data are expressed as frequencies (%).

**Results:**

Of the patients screened, 77 of 123 patients met inclusion criteria. Most patients were treated for a bone/joint infection (29/77, 38%). Ceftriaxone (18/82, 22%) and vancomycin (13/82, 16%) were the most frequently prescribed agents. The median DOT was 30 days (IQR 15, 42). On day of discharge, 52 opportunities for a pharmacist initiated intervention were identified with majority being clarifying DOT (19/52, 37%), streamlining or escalating antibiotic (8/52, 15%), and optimizing drug dose (8/52, 15%). OPAT-related discharge delays resulted in an excess of 58 hospital days and over 25% of patients (20/77) were readmitted 30 days after discharge. The most common post-discharge issues (n=56) were worsening infection (11/56, 20%), PICC line issues (9/56, 16%), and drug related adverse events (8/56, 14%).

**Conclusion:**

A pharmacist on a dedicated OPAT service can assist with antimicrobial selection, treatment duration, and drug monitoring to promote patient safety in patients discharged on antimicrobials.

**Disclosures:**

**All Authors**: No reported disclosures

